# ESTABLISHING LEVELS OF ARM–HAND ACTIVITIES IN STROKE PATIENTS: THE ARM-HAND-ACTIVITIES-SCALE (AHAS)

**DOI:** 10.2340/jrm.v58.44414

**Published:** 2026-01-21

**Authors:** Christina HEBENSTREIT, Miriam BINTER, Jörg WISSEL, Klemens FHEODOROFF

**Affiliations:** 1 KABEG Gailtal-Klinik Hermagor, Hermagor, Austria; 2 Neurologie und Psychosomatik am Wittenbergplatz, Berlin, Germany

**Keywords:** stroke, upper extremity paresis, arm–hand activities, FMA, ARAT, psychometrics: treatment strategies

## Abstract

**Objective:**

Post-stroke limitations in arm–hand activities are prevalent, yet a system for categorizing these limitations like the Functional Ambulation Categories (FAC) is lacking. The Arm-Hand-Activities-Scale (AHAS) was developed and psychometric properties of this classification were investigated.

**Design:**

Mixed methods to examine comprehensibility, inter-rater reliability, correlations with the Fugl-Meyer Assessment (FMA) and the Action Research Arm Test (ARAT), and sensitivity to change.

**Subjects/Patients:**

76 professionals answered a comprehensibility questionnaire and 85 professionals an inter-rater reliability questionnaire.

**Methods:**

For comprehensibility and inter-rater reliability, standardized video sequences of each category were assessed. Cut-off values were identified by comparing the AHAS categories with the FMA (*n* = 10) and the ARAT (*n* = 71). For sensitivity-to-change studies, 71 stroke patients were followed for 4 weeks of inpatient rehabilitation. Spearman correlation coefficients were calculated to examine the relationship between AHAS, FMA, and ARAT outcomes. A Wilcoxon signed-rank test was applied for sensitivity to change.

**Results:**

The results on comprehensibility of the AHAS categories and the FMA cutoff values were highly consistent. Inter-rater reliability was good to excellent. A significant and strong correlation was found between ARAT and AHAS. The Wilcoxon signed-rank test for sensitivity to change was also significant.

**Conclusion:**

The AHAS provides a quick and simple classification system to assess the severity of arm–hand activity limitations after stroke This may help in selecting appropriate treatment strategies.

## LAY ABSTRACT

Arm–hand use is limited in more than 65% of patients after stroke. Measuring activity is important for goal setting and treatment planning but can be time consuming and difficult to understand. The Arm-Hand Activities Scale was developed as an observational scale on 5 levels to classify arm–hand activities in a simple and comprehensible way. Here we investigated whether 2 or more people have the same understanding of the levels, whether they reach a similar rating, and whether this categorization is sensitive to changes in arm and hand activities. All tests achieved good to very good results. The Arm-Hand-Activities-Scale is therefore helpful in selecting appropriate treatments.

Arm–hand paresis is a frequent long-term consequence after stroke and affects more than 65% of stroke survivors. Sensorimotor and awareness deficits impact on arm and hand activities such as reaching, fixing, grasping and releasing objects, and fine arm and hand use. Depending on the initial degree of paresis and impaired control of voluntary movements, development of post-stroke spasticity (PSS), and sensory and awareness deficits, different pharmacological and non-pharmacological treatment strategies should be applied for regaining the best level of arm and hand activities after a comprehensive multidisciplinary and multimodal rehabilitation programme (1–3).

A number of measurement and assessment procedures have been established to record the degree of severity, both at the level of body functions such as the Fugl-Meyer Assessment (FMA) and at the level of activities such as the Action Research Arm Test (ARAT) (4–6). All of them need specific training and are time-consuming (7).

Until now, no simple, comprehensible, and validated classification of arm–hand activities like the Functional Ambulation Categories (FAC) for walking has been available (8, 9). Consequently, establishing agreed levels of arm and hand activities was found to be quite elaborative. A simple but validated categorization of arm and hand activities to assign the most appropriate treatment strategies according to current level of activity and to agreed time frames after stroke is warranted (10–12). To address this issue, we identified 5 arm and hand activity levels related to the ICF Activities and Participation domain “carrying, moving, and handling objects” (13): no activities, fixing objects, grasp/release, fine arm/hand use, and near normal arm/hand activities (Table I). To establish psychometric properties for these categories, we conducted a series of studies between 2018 and 2023.

**Table I:** Arm-Hand-Activities-Scale (AHAS)

Category	Description
No activities	No useful activities
Fixing objects	Arm/hand can be actively moved to a horizontal surface to fix objects in place
Grasp/release	Arm/hand can be actively moved to a horizontal surface; hand can perform grasping/releasing activities to hold larger objects in place
Fine arm/hand use	Arm can be raised against gravity; hand can be used for fine motor tasks
Near-normal arm/hand activities	Arm/hand can be used in bimanual tasks, despite mild coordination disorder, muscle tone fluctuations, and reduced speed

## METHODS

For testing the comprehensibility and inter-rater reliability of the proposed categories we developed a structured comprehensibility questionnaire in 3 steps. Instructions were closely related to the movement instructions of the Fugl-Meyer Assessment Arm Section (FMA-AS) and to specific arm–hand tasks. The final questionnaire included 24 questions covering expected agreement but also expected contradictions (Appendix S1). Participants also assessed 10 standardized video sequences with 2 videos per category showing patients with different FMA-AS scores (see video sequences – Supplementary videos 1–4).

Evaluation of ratings by professionals/patients was performed in 3 scenarios: for all responses; for instances where participants rated the videos as expected; and for instances where participants differed from the categorization intended in the video cases.

A sample size calculation indicated that a minimum of 60 respondents was required for the comprehensibility study and 76 respondents for the inter-rater reliability study to obtain significant results.

To identify cut-off values for the different categories, we compared the Arm-Hand-Activities-Scale (AHAS) estimates with the FMA-AS scores as a gold standard for assessing control of voluntary movements and with the ARAT scores as the gold standard for assessing levels of arm–hand activities (6).

Sensitivity to change was tested in a sample of subacute stroke patients, who undertook a comprehensive inpatient stroke rehabilitation programme and were followed for 4 weeks. Inclusion criteria for this study were patients between 18 and 80 years with a first ever stroke in the middle cerebral artery region in subacute stage according to Bernhard et al. (11). Furthermore, adequate language and task comprehension, adequate insight, and the capacity to focus attention for 30 min and to make decisions was required for this part of our study. Initial and follow-up rating was performed by the same rater.

Data analysis included demographic statistics of the professionals and the patients responding to the questionnaires and the sensitivity-to-change study (mean, standard deviation (SD), frequency and percentage). *P*-values were calculated where appropriate. To check for normal distribution of the metric variables a Kolmogorov–Smirnov test and a Shapiro–Wilk test were calculated.

To assess the comprehensibility of the AHAS categories, a Pearson χ^2^ test for subgroup analysis for differences between the individual categories of the AHAS and a Kruskal–Wallis rank sum test for testing the impact of independent group variables were used. To determine differences in responses between different professional groups and to assess the impact of professional experience, a Dunn’s Pairwise Comparison was conducted as a post-hoc analysis. In addition to the overall result, professional subgroups were evaluated using square-weighted kappas.

To check the correlation of the FMA-AS and the ARAT to the proposed AHAS categories, rank correlation coefficients (Spearman’s rho) were calculated. Sensitivity to change in the individual categories of the AHAS categories between t0 and t1 was tested with a Wilcoxon signed-rank test.

Ethical approvement by the Ethics Committee of the Province of Carinthia (Appendix S2) was obtained for all studies involving patients.

## RESULTS

Seventy-six individuals took part in the comprehensibility study (see Table II for demographics), achieving an accuracy rate of 94.7% (CI 87.1–98.5%). Participants on average achieved 21.2 ± 1.8 out of 24 correct answers, with a minimum score of 16 points.

**Table II:** Demographics for the AHAS comprehensibility study

Profession (*n* = 76)	No.
Physician experienced in rehabilitation	12 persons
Physiotherapists	16 persons
Occupational therapists	42 persons
Patients/relatives	6 persons
Professional experience (years)	
No experience	6
0–1 year	15
2–5 years	19
6–10 years	17
11–20 years	12
> 20 years	7

The comprehensibility was consistently high across different groups: patients and relatives scored 22.2 ± 1.2, physicians 21.8 ± 1.7, occupational therapists 21.1 ± 1.9, and physiotherapists 20.9 ± 1.9. There were no significant differences based on professional experience (Kruskal–Wallis test *p* = 0.229, supported by Dunn’s Pairwise Comparison; data not shown).

**Fig. 1:**
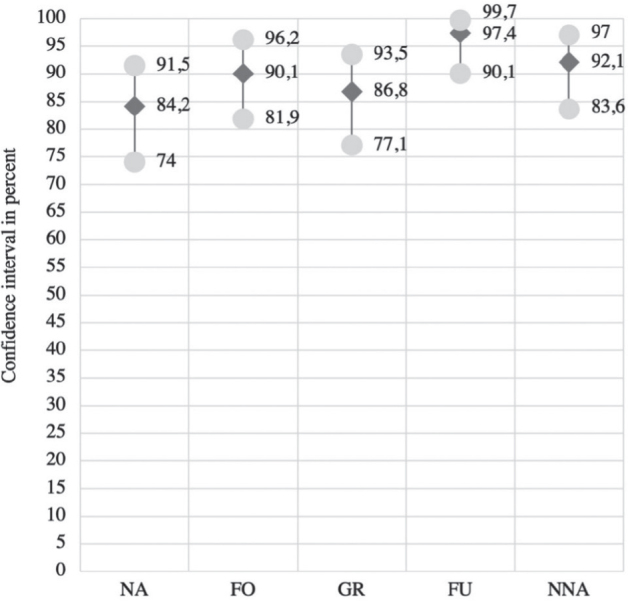
Degree of comprehensibility (all respondents; mean; min - max). NA: no activities, FO: fixing objects, GR: grasp/release, FU: fine arm/hand use, NNA: near-normal arm/hand activities.

The term “fine arm/hand use” had the highest comprehensibility at 97.4%, followed by “near-normal arm/hand activities” (92.1%), “fixing objects” (90.1%), “grasp/release” (86.8%), and “no activities” (84.2%) (Fig. 1).

In total, 85 individuals participated in the inter-rater reliability survey including 9 physicians (11%), 31 physiotherapists (36%), 33 occupational therapists (39%), and 12 stroke patients or relatives (14%) (see Table III for demographics).

**Table III:** Demographics for the inter-rater reliability survey

Profession (*n* = 85)	No.
Physician experienced in rehabilitation	9 persons
Physiotherapists	31 persons
Occupational therapists	33 persons
Patients/relatives	12 persons
professional experience (years)	
No experience	12
0–1 year	12
2–5 years	20
6–10 years	20
11–20 years	9
> 20 years	12

**Fig. 2:**
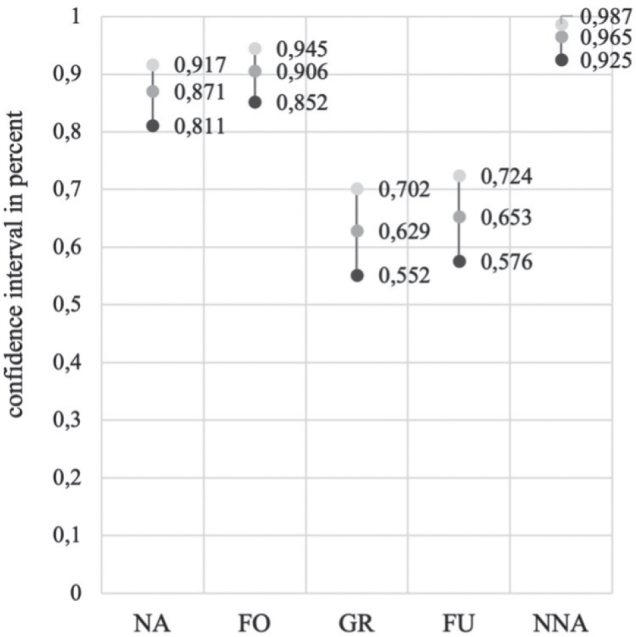
Confidence intervals of the responses across all professional groups. NA: no activities, FO: fixing objects, GR: grasp/release, FU: fine arm/hand use, NNA: near-normal arm/hand activities.

Cohen’s kappa for all respondents and all categories was 0.756. The confidence interval was 0.871 (0.811–0.917) for the “no activities” subgroup, 0.906 (0.852–0.945) for the “fixing objects” subgroup, 0.629 (0.552–0.702) for the “grasp/release” subgroup, 0.653 (0.570.724) for the “fine arm/hand use” subgroup, and 0.653 (0.576–0.724) for the “near normal arm/hand activities” subgroup (Fig. 2).

**Fig. 3:**
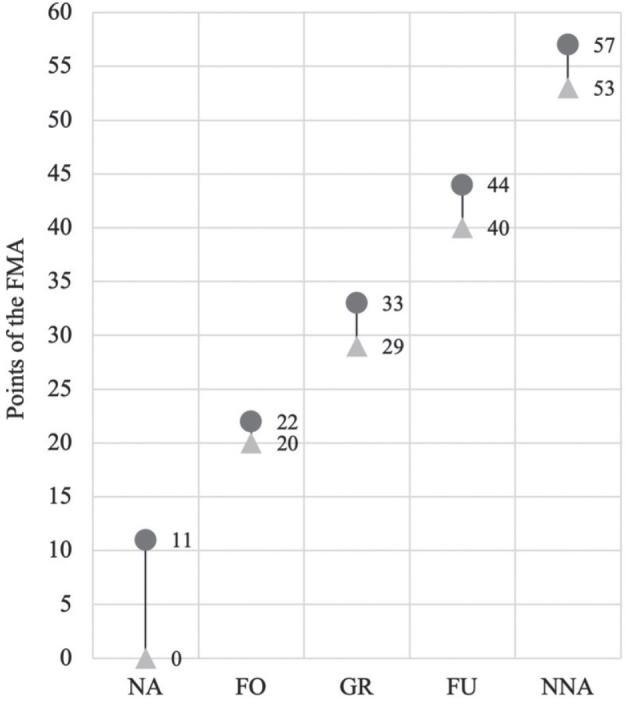
Range of values from the FMA for each value of the AHAS. Figures refer to correct answers. NA: no activities, FO: fixing objects, GR: grasp/release, FU: fine arm/hand use, NNA: near-normal arm/hand activities.

Descriptive statistics analysed FMA ranges for each AHAS value. The ranges are shown in Fig. 3. To test AHAS cut-off values by correlation with the FMA for the upper extremity, interrater reliability results were compared with patient FMA-AS scores from the standard video sequences. A significant correlation between AHAS values and FMA was observed (*p* = 0.92; *p* < 0.001). Incorrect questionnaire answers showed a medium-strong correlation (*p* = 0.60; *p* < 0.001), while correct answers had an even stronger correlation (*p* = 0.98; *p* < 0.001; data not shown). Significant differentiation of all subgroups was confirmed using the Kruskal–Wallis test (*p* = 0.0001) and post-hoc using the Dunn test (*p* = 0.0000). Statistical anomalies arose in evaluating incorrect answers, specifically between “grasp/release” and “fine arm/hand use” (*p* = 0.0984) or “grasp/release” and “near-normal arm/hand activities” (*p* = 0.1089).

**Fig. 4:**
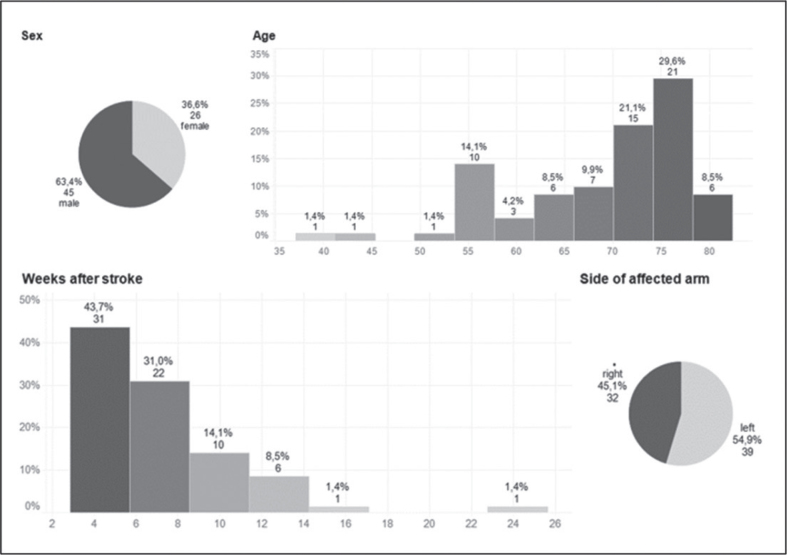
Patient demographic: AHAS–ARAT correlation and sensitivity to change.

A validation study confirmed the correlation with the ARAT and demonstrated sensitivity to change. The study included 71 patients: 45 men (63.4%) and 26 women (36.6%), as detailed in Fig. 4.

**Fig. 5:**
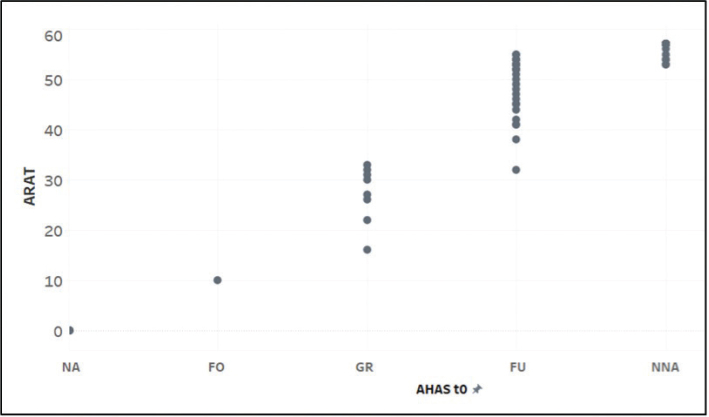
Scatterplot between AHAS and ARAT sum score. AHAS: Arm-Hand-Activities-Scale; ARAT: Action Research Arm Test; NA: no activities, FO: fixing objects, GR: grasp/release, FU: fine arm/hand use, NNA: near-normal arm/hand activities; t0: baseline.

All participants completed the study. Spearman’s correlation revealed a strong and significant relationship (*r* = 0.926; *p* < 0.001) between the total ARAT score and the AHAS, indicating that higher ARAT values correspond to higher AHAS categorization (Fig. 5).

**Fig. 6:**
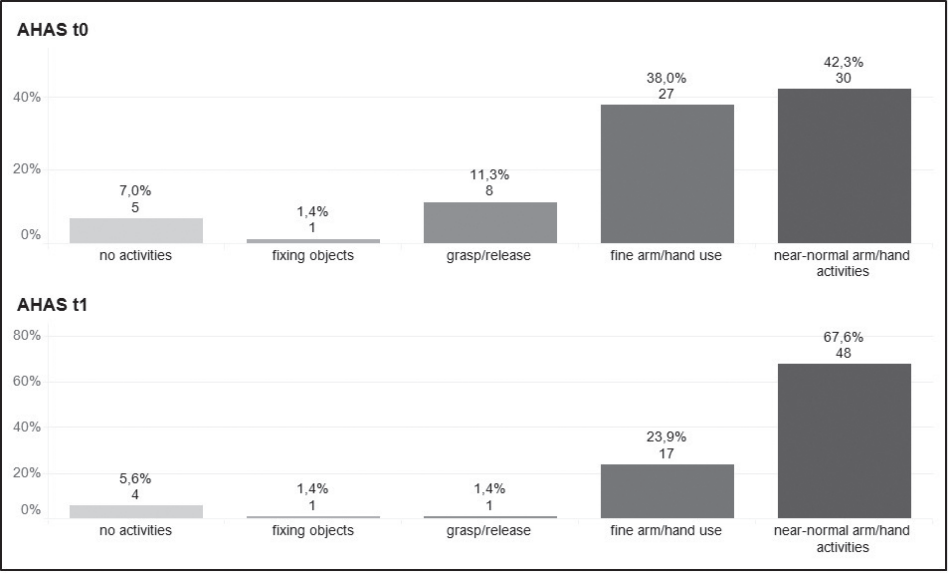
Sensitivity to change from baseline to follow-up. AHAS: Arm-Hand-Activities-Scale; t0: baseline; t1: 4-weeks follow-up.

The Wilcoxon signed-rank test was employed to assess sensitivity to change, demonstrating a significant difference (*z* = –5.29; *p* < 0.001) (24, 28). At t1, 28 out of 71 patients (39.4%) reached a higher category, with no participant’s rating being worse than at t0 (Fig. 6).

## DISCUSSION

Assessing individual levels of functioning is a key feature for goal-oriented neurorehabilitation and intervention planning in a multimodal approach. According to the International Classification of Functioning, Disability and Health (WHO-ICF), both, the level of body function (i.e., control of voluntary movements) and the level of activities/participation (i.e., reaching, fixing, grasping and releasing objects, fine arm and hand use) should be covered.

In a comprehensive review by Duncan Millar et al. (7) on outcome measures in arm post-stroke rehabilitation trials, encompassing 243 studies on methods for recording and assessing the severity of arm–hand paresis, 144 distinct measurement instruments were identified. Among these, the FMA was the most frequently utilized tool (33%), followed by the ARAT (23%), and the Modified Ashworth Scale (mAS) (also at 23%). Additionally, 66 different aspects related to arm–hand paresis after stroke were identified as significant for both, affected individuals and their caregivers. These aspects include difficulties in daily routines, the need for assistance, the ability to work and drive, and emotional considerations. Common activities of daily living (ADL) scales, such as the Barthel Index (14) or the Functional Independence Measure (15), capture limitations in selected self-care activities, such as toileting, feeding, dressing, and grooming, but do not rate limitations in arm and hand activities in detail. Covering all these aspects may not be feasible in daily routine.

Sabari et al. (16) noted that the FMA requires an assessment time of up to 30 min. The ARAT also takes at least 20–30 min to complete; therefore, both are rarely used in routine clinical practice.

Self-reports on arm-hand activities such as the ArmA scale (17, 18) or the Disability Assessment Schedule (DAS) (19) have been suggested for recording task performance and for choosing appropriate treatment pathways, including spasticity management with Botulinum Toxin A and non-pharmacological interventions such as task-oriented training. However, these self-reports should be validated by objective tests to measure capacity or by observing performance during daily routines.

According to Tyson (20), stroke service users require clear and understandable information regarding assessment purposes and consistent, objective feedback regarding their progress in simple terms. Hence, the clarity of these terms is essential. Ideally, individual items on a scale can be used directly for understandable, person-centred goal setting and for goal attainment scaling.

The AHAS, an observational classification scale, includes 5 categories related to daily activities such as “fixing objects”, “grasp/release”, and “fine arm/hand use”. This aligns with the WHO-ICF Activities domain (d430-d449 – carrying, moving, and handling objects) and supports task-oriented training. For research, AHAS can help form homogeneous groups.

According to Rohrmann, a rating scale with 5 categories is considered most effective, as the risk of incorrect allocation increases if there are too many options available. It is important that the categories are as equidistant as possible and that the wording is easy to remember (21). Consequently, the AHAS was designed as a 5-point scale, even though the FAC, which served as a model, has 6 levels for more precise subdivision. The AHAS has a clear delineation of categories between the individual FMA-AS scores with no overlapping (see Fig. 3, Table IV).

**Table IV:** FMA cutoff values with tasks in cut-off area, sub- and superordinate tasks in relation to AHAS categories

	Cut-off values	Tasks in the cut-off area	Subordinate tasks	Superordinate tasks
No activities	0–11 points	elbow flexion shoulder adduction with internal rotation finger mass flexion scapular elevation finger mass extension (relaxation of flexion)		cylindrical grasp
Fixing objects	20–22 points	shoulder abduction hand to lumbar spine	elbow extension	shoulder flexion to 90°, elbow extended
Grasp/release	29–33 points	palmar prehension wrist flexion/extension, elbow at 90°	scapular retraction	wrist stable, elbow at 90°
Fine arm/hand use	40–44 points	shoulder abduction to 90°, elbow extended forearm supination movement with normal speed	movement without dysmetria	wrist stable, elbow extended
Near-normal arm/hand activities	53–57 points	spherical grasp shoulder flexion to 180°, elbow extended hook grasp wrist circumduction	lateral prehension	

Max FMA-AS sum score = 60 points (no reflex activities).

**Fig. 7:**
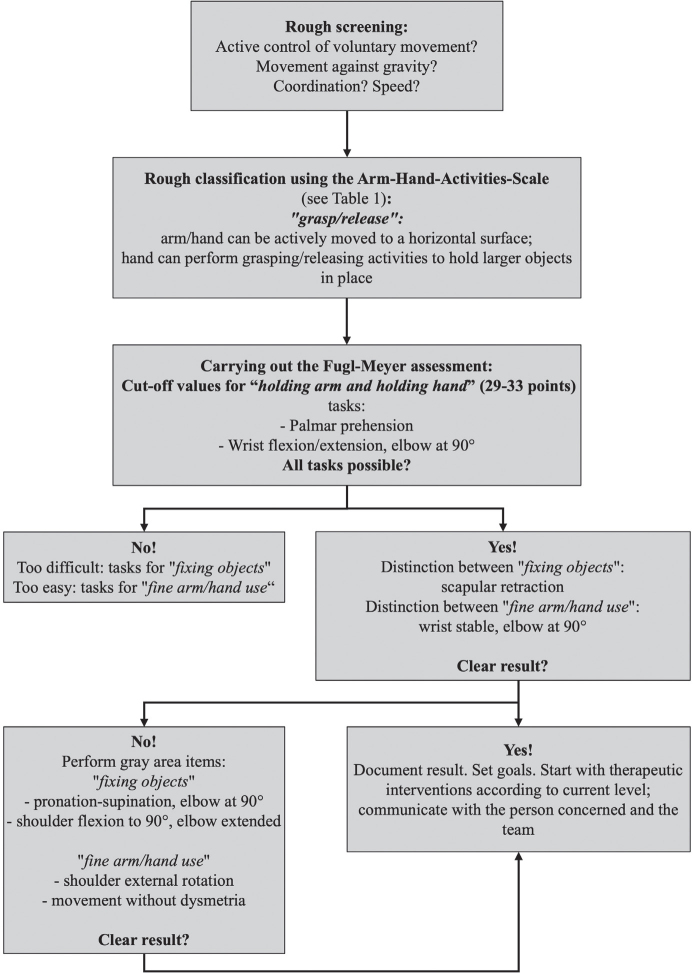
Suggested algorithm for using the Arm-Hand-Activities-Scale.

The results of the comprehensibility questionnaire demonstrated a high level of understanding the concepts in various professions regardless of their length of clinical experience. Consequently, it can be concluded that the AHAS is suitable for diverse user groups. One notable strength of the AHAS is its simplicity for patients and their relatives. Categorizing the arm–hand activities with the AHAS takes around 5–8 min (Fig. 7).

The interrater reliability tests showed excellent agreement (κ > 0.8) for “no activities” and “fixing objects”, and good agreement (κ > 0.6) for “fine arm/hand use”, “near normal arm/hand activities”, and “grasp/release”. The latter categories may need further clarification.

In the correlation study with the ARAT, more patients were in the 2 upper categories, “fine arm/hand use” and “near-normal arm/hand activities”, than in the lower categories, “no activities” and “fixing objects. This is because of the inclusion criteria, which demanded adequate language and task comprehension, adequate insight and the capacity to focus attention for 30 min and to make decisions.

A possible explanation for the overlaps of the categories “grasp/release” with “fine arm/hand use” and near-normal arm/hand activities” (see Fig. 5) may be due to the different construction of the tests. The ARAT measures at the level of capacity (i.e., what a person can do), while the AHAS measures the average effort in everyday life by observation (i.e., performance – what a person does in real life). It is important to note that the ARAT instructions make demands not only on motor components but also on attention, concentration, visual, auditory, and sensorimotor perception. Consequently, short-term capacity may exceed performance during daily routine. Patients may therefore achieve a higher score on the ARAT but a lower score on the AHAS. Learned non-use must also be considered as a potential cause for reduced arm/hand activities in daily routine. However, this supports the case to emphasize task-oriented training.

### Limitations

As an observational scale, the AHAS is not a measurement but a categorical classification. This can lead to subjective and comprehensibility-related uncertainties. Therefore, further studies should reveal the need for specific training material in different languages.

Because the AHAS relies on observation, some subjectivity remains unavoidable. Standardized procedures should be used to create a standardized assessment framework similar to that used for preparing the video sequences for the comprehensibility and interrater reliability studies. Patients should be observed when carrying out various tasks as part of their daily routine.

Although the sample size calculation to obtain significant results has been exceeded in all studies, results should be confirmed by studies with large sample sizes and similar inclusion criteria.

Currently, psychometric criteria on comprehensibility to the full extent have only been carried out in the German language. The comprehensibility questionnaire has been exclusively designed for the purpose of this study, as a corresponding counterpart could not be found in the literature (Supplementary data 1). Due to the positive results from the comprehensibility analysis, it is now available for future research. For further dissemination and implementation of the AHAS in acute care and in rehabilitation centres, comprehensibility testing in all major languages is warranted. The comprehensibility testing for the English language is on the way.

### Conclusion

The AHAS provides a quick and simple classification system for everyday clinical practice to assess the degree of arm/hand activity limitations in stroke survivors. As an observational scale, the AHAS captures what patients actually do (ICF-performance qualifier). A shared decision by the team is helpful to prevent misallocations and to promote a task-oriented training of arm-hand activities. It also enables a rapid referral to appropriate treatment pathways, such as spasticity management and task-oriented training. In the context of clinical research, the AHAS could be helpful in establishing homogenous groups to avoid selection biases and to change view on active / passive arm and hand functions.

## Supplementary Material










