# Supplementary material for: ESTABLISHING LEVELS OF ARM–HAND ACTIVITIES IN STROKE PATIENTS: THE ARM-HAND-ACTIVITIES-SCALE (AHAS)
Source: J Rehabil Med. 2026 Jan 21;58:44414. doi: 10.2340/jrm.v58.44414 (PMC12848808; doi:10.2340/jrm.v58.44414)
Supplement: Supplementary file 1 [file JRM-58-44414-s1.pdf]

Supplementary material has been published as submitted. It has not been copyedited, or typeset by Acta Dermato-Venereologica

Appendix S1. Questionnaire for evaluation the comprehensibility of the AHAS (agree / disagree / don`t know)

1. If the hand is affected by a mass flexion (uncontrollable mass movements when bending), the hand does not have any everyday activities and is therefore regarded as having "*no activity*".
2. A person with "*fine arm/hand use"* is able to move the affected arm against gravity while standing.
3. A person who can use the affected arm/hand for *"fixing objects"* is able to hold a bottle of shower gel while taking a shower.
4. A person with a *"near-normal hand activity"* must not exhibit any restrictions/limitations.
5. A person who can use the affected hand for *"grasp/release"* can fix a piece of paper on a stable surface.
6. *"No activity"* means that controlled voluntary motor functions (movements that are caused and controlled by volition or by conscious efforts) are possible.
7. A person who can use the affected arm/hand for *"grasp/release"* can carry a cloth bag with their bended (flexed) elbow for longer than a minute.
8. A person who can use the affected arm/hand for *"fixing objects"* in place can **passively** position the affected arm/hand on a table while sitting.
9. An arm or a hand with no usable functions is regarded as having *"no activity"* for everyday activities.
10. A person with *"fine arm/hand use"* can perform tasks on a stable support surface.
11. A person with a *"near-normal hand activity"* can carry a shopping bag of about 5 kg using the affected hand without support.
12. A person with *"fine arm/hand use"* can perform bimanual tasks.
13. For a person with mild coordination disorders of the affected arm/hand, the *"near-normal hand activity"* is chosen nonetheless for the activity scale.
14. A person who can use the affected arm for *"fixing objects"* can tuck a newspaper under their affected arm and hold it in place for more than a minute.
15. A person who can use the affected arm/hand for *"grasp/release"* can perform tasks above chest level.
16. Bimanual tasks can be performed using a *"fine arm/hand use"* as an assist.
17. Bimanual tasks such as driving in a nail are only possible with assistance for a person with a *"near-normal arm/hand activity"*.
18. A hand is regarded as having *"no activity"* if it can perform only mass extension movements (uncontrollable mass movements when extending) but does not have any usable everyday activities.
19. A person who can use the affected arm/hand for *"fixing objects"* in place can grasp and release a bottle.
20. A person who can use the affected arm/hand for *"fixing objects"* in place can fix a piece of paper in place for longer than a minute.
21. A person who can use the affected arm/hand for *"grasp/release"* can roughly control the muscle tone.
22. A person with *"fine arm/hand use "* can move the affected arm/hand without help to the non-affected side.
23. A person who can use their hand for *"grasp/release"* shows signs of a grasping function.
24. A person with a *"near-normal arm/hand activity"* can perform targeted movements.

Fig S1. Flow-Chart of patient recruitment for the ARAT correlation study

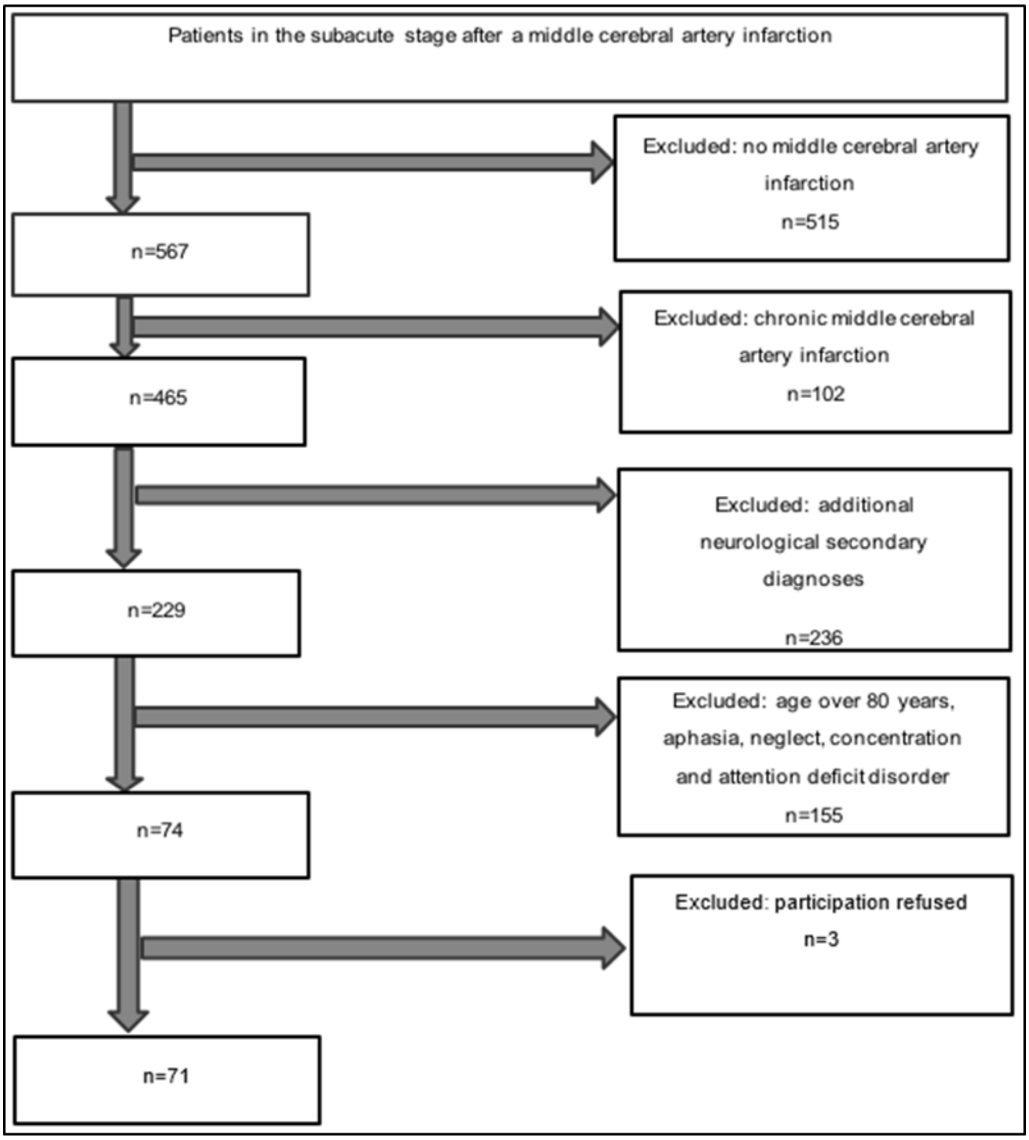

Appendix S2. Ethics votes of the Ethics Committee of the Province of Carinthia (German)

ethikkommission kärnten

VOTUM

(Lokale Ethikkommission)

Gültig bis 26.01.2020

**EK-Nummer:**	**A 32/18**
**Studientitel:**	Überprüfung der Verständlichkeit und internen Reliabilität der Arm Hand-Aktivitätsskala (AHAS)
**Studienkurzname:**	-
**EudraCT-Nr:**	-
**Antragsteller:**	BSc Miriam Berger
**Institution(en):**	KABEG Gailtal Klinik
**Sponsor:**	-
**Prüfzentrum/ Prüfzentren:**	KABEG Gailtal Klinik
**Präsentator der Studie**	BSC Miriam Berger

Die o.a. Studie wurde in der Sitzung der Ethik-Kommission am 12.12.2018 behandelt.

Die Ethikkommission ist zu folgendem Schluss gekommen:

Es besteht kein Einwand gegen die Durchführung dieser Studie in der vorliegenden Form.

Dieses Votum gilt **für ein Jahr ab dem Datum der Ausstellung des Votums.** Bei längerer Studiendauer ist rechtzeitig vor Ablauf der Gültigkeit des Votums ein Zwischenbericht vorzulegen (**Formular „ Studienbericht"**) um eine etwaige Verlängerung zu erlangen.

Stimmberechtigte Mitglieder bei der Behandlung waren: Siehe Anwesenheitsliste der Sitzung.

Kommissionsmitglieder, die für diesen Tagungsordnungspunkt als befangen anzusehen waren und daher gemäß Geschäftsordnung an der Entscheidungsfindung und Abstimmung nicht teilgenommen haben: keine

Das Votum der Ethik-Kommission berührt in keiner Weise die alleinige Verantwortung des Antragstellers für die Durchführung der Studie.

Ethikkommission des Landes Kärnten

VORSITZENDER:
Univ.-Doz. Dr. H.-J. Gallowitsch

SEKRETARIAT:
Sandra Veratschnig
c/o ELKI, Klinikum Klagenfurt
Feschnigstraße 11
9020 Klagenfurt

TEL. 0463 538-25402
MAIL sandra.veratschnig@kabeg.at
office@ethikkommission-kaernten.at
WEB www.ethikkommission-kaernten.at

LAND KÄRNTEN

ethikkommission kärnten

Vorsitzender: Dr. med. univ. Martin C. Spendel
Geschäftsstelle: Natasche Jung +43 463 55212-50195
Klinikum Klagenfurt | Feschnigstraße 11
9020 Klagenfurt am Wörthersee | Austria
office@ethikkommission-kaernten.at
www.ethikkommission-kaernten.at

VOTUM

EK-Nummer	S2022-20
EudraCT-Nummer	-
Projekttitel	Validierung der Arm-Hand Aktvitätsskala (AHAS) anhand des Action-Research-Arm Test (ARAT)
Prüfplan-Code	AHAS-GTK_2022-01
Prüfer	Christina Hebenstreit, BSc
Prüfzentrum	KABEG Gailtal-Klinik
Antrag vom	2022-08-29
Eingang am	2022-10-02
Sponsor	-
Antragsteller	Christina Hebenstreit, BSc
Institution	KABEG Gailtal-Klinik

Personenbezogene Ausdrücke in diesem Dokument umfassen jedes Geschlecht gleichermaßen.

Sehr geehrte Frau Hebenstreit,

die Ethikkommission des Landes Kärnten hat das o. g. Projekt in ihrer Sitzung am 2022-09-14 im ordentlichen Verfahren beraten und fasst folgenden

Beschluss:

Aus forschungsethischer und berufsrechtlicher Sicht werden keine Bedenken erhoben.

Begründung:

1. Der Antragsteller legt am 2022-10-02 revidierte Dokumente vor. Diese Dokumente erfüllen die im konditionalen Votum vom 2022-09-14 genannten Auflagen und werden von der Ethikkommission akzeptiert.
2. Es handelt sich um eine relevante Fragestellung, die mit geeigneter Methodik beantwortet werden soll.
3. Die vom Antragsteller vorgenommene Bewertung des Nutzen-Risiko-Verhältnisses ist plausibel.
